# Preoperative brain MRI for clinical stage IA lung cancer: is routine scanning rational?

**DOI:** 10.1007/s00432-018-2814-2

**Published:** 2018-12-07

**Authors:** Lingdun Zhuge, Yangle Huang, Shengfei Wang, Juntao Xie, Binhao Huang, Difan Zheng, Shanbo Zheng, Yue Zhao, Hengyu Mao, David O. Wilson, James D. Luketich, Jiaqing Xiang, Haiquan Chen, Jie Zhang

**Affiliations:** 1 Department of Thoracic Surgery, Fudan University Shanghai Cancer Center, 270 Dong-An Road, Shanghai, 200032 China; 2 Department of Oncology, Shanghai Medical College, Fudan University, Shanghai, 200032 China; 3 Division of Pulmonary, Allergy and Critical Care Medicine, Department of Medicine, University of Pittsburgh, Pittsburgh, PA USA; 4 Department of Cardiothoracic Surgery, University of Pittsburgh Medical Center, Pittsburgh, PA 15213 USA

**Keywords:** Lung cancer, Brain MRI, Brain metastases

## Abstract

**Purpose:**

Early detection and control of lung cancer brain metastases (BMs) are important. However, several guideline recommendations are inconsistent with regard to routine preoperative brain MRI, especially in patients with clinical stage IA lung cancer. Our study evaluated the value of preoperative brain MRI in patients with clinical stage IA lung cancer.

**Methods:**

A retrospective analysis of patients with lung cancer was performed using a prospectively collected database. Clinical data and the results of brain MRI were collected and analyzed.

**Results:**

Patients with pathologically proved primary lung cancer who underwent an MRI at initial diagnosis were identified (3392 patients). In total, 170 patients (5.0%) were diagnosed with BMs. The increased frequency of BMs was significantly associated with advanced clinical stage (*P* = 0.000) and pathological type (*P* = 0.011). BMs were detected in 11 out of 1595 patients with clinical stage IA lung cancer (0.7%). BMs were more common in patients with clinical stage cT1c lung cancer (1.9%) than those with clinical stage cT1a or cT1b (0.1%, odds ratio = 21.30, 95% confidence interval: 2.7–166.9, *P* = 0.000). All patients with stage IA lung cancer and BMs had solid lung lesions (*P* = 0.002).

**Conclusions:**

Preoperative brain MRI might help identify BMs in patients with lung cancer that has progressed beyond stage IA. In patients with clinical stage IA lung cancer, we do not recommend preoperative brain MRI, but it may potentially be beneficial in those with solid T1c cancers.

## Introduction

Lung cancer is the leading cause of cancer-related death worldwide (Siegel et al. 2014). Brain, liver, adrenal glands, and bone are the most frequent extrathoracic sites for lung cancer metastases and have a significant, negative prognostic impact (Ampil et al. 2007; Sanchez de Cos et al. 2009). Occult extrathoracic metastasis may result in early post-operative recurrence and poor prognosis. Thus, detection of distant metastases when staging lung cancer is critical to avoid unnecessary surgery and initiate suitable multidisciplinary treatment.

Several studies indicated that 10–36% of lung cancer patients developed brain metastases (BMs) over the course of the disease (Schouten et al. 2002; Villano et al. 2015). BMs usually lead to higher mortality and decreased quality of life, with a median survival after diagnosis of less than 1 year.(Enders et al. 2016; Penel et al. 2001) It is estimated that 30–50% of lung cancer patients die from neurologic causes.(Nguyen and DeAngelis 2007) Therefore, early detection and treatment of BMs is of great importance. Magnetic resonance imaging (MRI) is the first imaging choice for diagnosing intracranial metastasis due to its improved sensitivity as compared with computed tomography (CT) and positron emission tomography (PET). MRI brain imaging has a reported sensitivity and specificity of 97.7% and 100%, respectively.(Kim et al. 2005) MRI brain imaging is superior to CT of the head for detection of small (< 1 cm) metastases, posterior fossa lesions, and multiple metastases (Purandare and Rangarajan 2015).

In early stage lung cancer, particularly in stage IA lung cancer, the prevalence of BMs at initial diagnosis varied among different reports, ranging from 1 to 18.9% (Jena et al. 2008; Na et al. 2008; Silvestri et al. 2013). In addition, 6.3–15.6% of patients developed BMs after surgery with curative intent (Hubbs et al. 2010; Nelson et al. 2011; O’Dowd et al. 2014). As a result, guideline recommendations are inconsistent with regard to whether preoperative cranial imaging should be routine in patients with early stage lung cancer. The National Comprehensive Cancer Network (NCCN) guidelines recommended brain imaging with MRI in patients with stage II to IV non-small cell lung cancer (NSCLC) (National Comprehensive Cancer Network 2018). In contrast, in the United Kingdom, the recommendations of the National Institute of Health and Care Excellence (NICE) (Baldwin et al. 2011) and the British Thoracic Society (Lim et al. 2010) are that all patients being considered for surgery with curative intent should receive routine brain imaging, regardless of clinical stage. The Choosing Wisely campaign, in combination with the Society of Thoracic Surgeons (STS) in the United States, released a recommendation that patients with suspected or biopsy-proven stage IA NSCLC do not require preoperative brain imaging unless they have neurological symptoms (The Society of Thoracic Surgeons 2013). No recommendations are universally applicable in clinical practice (Balekian et al. 2016). Additionally, due to the limited number of high-quality studies on BMs in patients with stage IA lung cancer, the level of evidence to support the current recommendations is relatively low. It is still controversial whether MRI brain imaging should be routinely performed for patients with stage IA lung cancer.

Our group previously analyzed the usefulness of bone scintigraphy and bronchoscopy in patients with early stage lung cancer (Li et al. 2015; Zhang et al. 2015). We performed the current study to evaluate the value of preoperative MRI brain imaging in lung cancer patients, especially those with clinical stage IA cancer. The aims of this study were to determine the incidence of BMs in patients with clinical stage IA lung cancer, to understand the association of BMs with other clinical features, and to evaluate the efficiency of preoperative MRI brain imaging in patients with early stage lung cancers.

## Materials and methods

### Patients

**Fig. 1:**
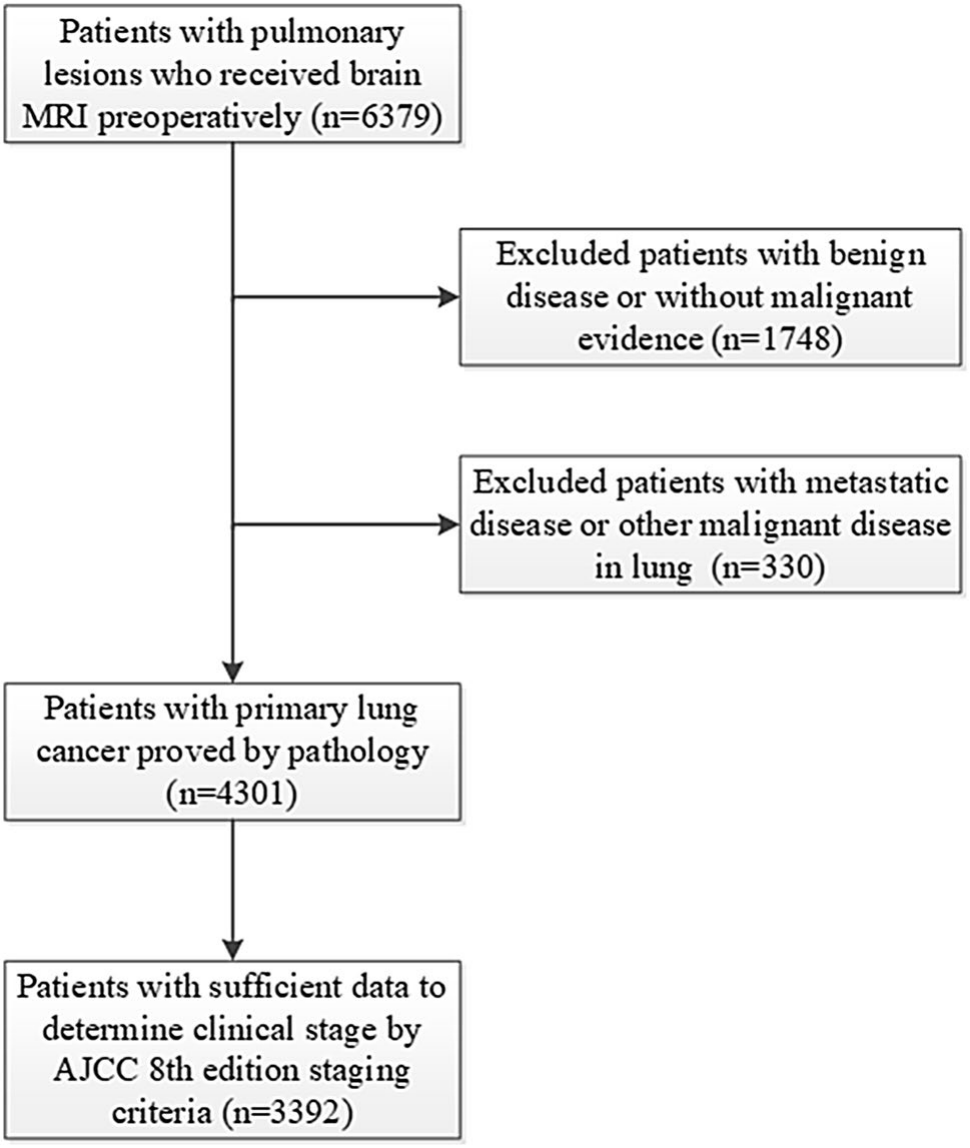
Criteria for selection and exclusion of research subjects

Patients with pathologically proved primary lung cancer who received MRI brain imaging at least once were identified in a retrospective analysis of a prospectively collected database containing both inpatients and outpatients with lung cancer treated by the Department of Thoracic Surgery, Fudan University Shanghai Cancer Center from January 2007 to December 2016. The needle biopsy or surgical pathology reports were reviewed by two independent pathologists. The clinical stage was based on the results of all preoperative examinations including clinical examination, blood tests, MRI brain, CT chest, ultrasound of the neck and abdomen, fiberoptic bronchoscopy, and bone scintigraphy. The 8th edition guidelines of the American Joint Committee on Cancer (AJCC) for lung cancer were applied for staging (Goldstraw et al. 2016). Clinical data were collected on age, sex, smoking history, tumor histologic type, and tumor biomarkers. Clinical stage (brain metastases excluded) and clinical data were used to analyze the risk factors for BMs. For patients with cT1N0 lung cancer, the lung lesions were characterized into solid nodules, mixed ground glass opacity (GGO), and pure GGO according to the preoperative CT chest imaging, and the latter two were described as nodules with a GGO component in the study. The inclusion criteria were: (1) older than 18 years; (2) diagnosed with primary lung cancer with pathological evidence; (3) received brain MRI examination at the initial diagnosis; and (4) sufficient information available for clinical staging (Fig. 1).

In patients with cT1N0 lung cancer with BMs, therapeutic strategies were modified. Suitable multidisciplinary therapy was performed to treat the metastatic disease. Surgical resection of the primary cancer in the lung followed, if appropriate.

### MRI brain

At our institution, all patients diagnosed with lung cancer were recommended to receive MRI brain imaging. The MRI protocol comprised sagittal T1-wighted Fluid-attenuated inversion recovery (FLAIR) imaging (repetition time (TR)/time to echo (TE), 2025/9.4; matrix, 320 × 192; field of view, 24 cm; slice thickness, 5 mm), axial T2-weighted FLAIR images (TR/TE, 8500/155; matrix, 256 × 192; field of view, 24 cm; slice thickness, 6 mm) and axial T2-weighted fast spin echo (FSE) images (TR/TE, 3660/102; matrix, 320 × 224; field of view, 24 cm; slice thickness, 6 mm). Gadopentetate dimeglumine (15 ml; 7.04 g) was used as a contrast agent at a rate of 1.5 ml/s. The post-contrast images included sagittal T-1 weighted fast spoiled gradient-echo (FSPGR) imaging (TR/TE, 210/2.3; matrix, 384 × 224; flip angle, 80°; field of view, 24 cm; slice thickness, 5 mm) and axial T1-weighted FSPGR images (TR/TE, 130/2.3; matrix, 384 × 224; flip angle, 85°; field of view, 24 cm; slice thickness, 6 mm).

### Statistical analysis

Statistical analyses were performed using SPSS (version 19.0, SPSS, Inc, Chicago, Ill). Continuous variables are presented as mean ± SD, categorical variables are presented as frequency and percentage. In univariate analysis, continuous variables were compared using an independent Student’s *t* test, and categorical variables were analyzed using Pearson *χ*^2^ test and the Fisher’s exact test, as appropriate. All variables found to be statistically significant were included in a logistic regression model to identify the independent predictors and risk factors for BMs.

## Results

After screening, 3392 eligible patients were included in this study (Fig. 1). Their clinical characteristics, including age, sex, clinical stage, smoking history, tumor biomarkers, and tumor histological type, are presented in Table 1.

**Table 1:** Patient clinical characteristics

Characteristic	*N* (%)^a^
Age, in median years (range)	61 (19–85)
Clinical T stage	
T1	2007 (59.2)
T2	892 (26.3)
T3	331 (9.7)
T4	162 (4.8)
Clinical N stage	
N0	2258 (66.6)
N1	361 (10.6)
N2	598 (17.6)
N3	175 (5.2)
Clinical M stage^b^	
M0	3155 (93.0)
M1	237 (7.0)
Clinical stage	
IA	1595 (47.0)
T1a	390 (11.5)
T1b	689 (20.3)
T1c	516 (15.2)
IB	250 (7.4)
II	534 (15.7)
III	776 (22.9)
IV	237 (7.0)
Sex	
Male	1899 (56.0)
Female	1493 (44.0)
Smoking history^c^	
Never	1828 (58.8)
Ever	1280 (41.2)
Pathology	
Adenocarcinoma	2374 (70.0)
Squamous cell carcinoma	609 (18.0)
Other	409 (12.0)

^a^Unless otherwise specified

^b^Distant metastasis other than brain

^c^Smoking history was not available for 284 patients (8.4%)

### MRI brain imaging

BMs were detected by preoperative MRI brain imaging in 170 patients (5.0%), including 87 (51.2%) with a single metastatic lesion and 83 (48.8%) with multiple metastases to the brain. Neurologically asymptomatic metastases were found in 149 of the 170 patients (87.6%); only 21 patients (12.4%) had neurologic symptoms. The BMs frequency per clinical T stage (T1, T2, T3 and T4) was 1.9%, 8.5%, 11.2% and 11.1%, respectively. Univariate analysis revealed that BMs were associated with clinical stage, smoking history, tumor histologic type, carcinoembryonic antigen (CEA) level, cytokeratin 19 fragment (CYFRA) 21-1 level, and cancer antigen (CA) 125 level (Table 2). In multivariate analysis, increased frequency of BMs was significantly associated with advanced clinical stage and histologic type other than squamous cell carcinoma. Compared with clinical stage IA lung cancer, the BMs were more likely to occur in patients with stage IB (OR 7.0, *P* = 0.007), stage II (OR 7.9, *P* = 0.001), stage III (OR 18.3, *P* = 0.000) and stage IV (OR 56.6, *P* = 0.000) lung cancer (Table 2).

**Table 2:** Clinical features and their correlation with brain metastasis

Clinical feature	*N* (%)^a^	Univariate analysis			Multivariate analysis		
		OR	95% CI	*P* value	OR	95% CI	*P* value
Clinical stage				0.000			
IA	11 (0.7)	Reference	–	–	Reference	–	–
IB	5 (2.0)	2.938	1.012–8.530	0.047	6.962	1.706–28.411	0.007
II	19 (3.6)	5.313	2.512–11.238	0.000	7.906	2.354–26.547	0.001
III	68 (8.8)	13.831	7.270–26.311	0.000	18.295	6.105–54.831	0.000
IV	67 (28.3)	56.753	29.422–109.471	0.000	56.646	18.163–176.660	0.000
Pathology				0.000			0.024
Adenocarcinoma	117 (4.9)	Reference	–	–	Reference	–	–
Squamous cell carcinoma	9 (1.5)	0.289	0.146–0.573	0.000	0.260	0.099–0.687	0.007
Other	44 (10.8)	2.325	1.616–3.346	0.000	0.946	0.487–1.836	0.869
Age (≤ 60/> 60)	91 (5.4)/79 (4.6)	0.837	0.615–1.141	0.261	–	–	–
Sex (male/female)	103 (5.4)/67 (4.5)	1.221	0.890–1.673	0.215	–	–	–
Smoking history (never /ever)	46 (2.5)/62 (4.8)	1.972	1337–2.908	0.001	–	–	–
CEA (< 5 ng/ml /≥5 ng/ml)^b^	34 (1.6)/51 (7.6)	4.941	3.172–7.695	0.000	–	–	–
CYFRA21-1 (< 3.3 ng/ml /≥3.3 ng/ml)^c^	27 (1.7)/56 (4.7)	2.809	1.763–4.475	0.000	–	–	–
CA125 (< 35 U/ml /≥35 U/ml)^d^	46 (2.0)/23 (8.4)	4.569	2.724–7.664	0.000	–	–	–

*CA* cancer antigen, *CEA* carcinoembryonic antigen, *CYFRA* cytokeratin 19 fragments, *CI* confidence interval, *OR* odds ratio

^a^Percentages are based on the total number of patients with each characteristic in the study group

^b^Data on CEA levels was absent in 632 patients (18.6%)

^c^Data on CYFRA21-1 was absent in 651 patients (19.2%)

^d^Data on CA125 was absent in 768 patients (22.6%)

### Treatment

Therapeutic strategies were altered in 149 patients due to the positive finding of BMs by MRI preoperatively. Surgery was canceled in 87 patients (58.4%) and altered from curative intent to palliative resection in 18 patients (12.1%). Brain radiotherapy was performed in 42 patients (28.2%), including 6 patients who underwent stereotactic radiosurgery, while brain surgery was conducted to resect the metastatic lesions in 3 patients (2.0%). Additional chemotherapy or chemoradiotherapy was given to 10 patients (6.7%).

### Risk factors of BMs in patients with clinical stage IA lung cancer

Because the risk factors for BMs in lung cancer patients with clinical stage IA cancer had not been widely identified, we performed a more detailed analysis to analyze the risk factors and determine the appropriateness of preoperative MRI brain imaging.

Of the 1595 patients with stage IA lung cancer, BMs were detected by MRI in 11 (0.7%) including 1 patient (0.1%) with clinical stage T1b cancer and 10 of 516 patients with stage T1c lung cancer (1.9%). Univariate analysis revealed that the risk of BMs was higher in clinical stage T1c lung cancer than T1a and T1b ones (OR 21.3, *P* = 0.000) (Table 3). Furthermore, to estimate the relative risk of BMs in T1cN0 lung cancer, the difference in the incidence BMs was compared between patients with T1cN0 tumors and clinical stage IB tumors. The odds of BMs were similar in these two patient subsets. The frequency of BMs was 1.9% in patients with T1cN0 cancer and 2.0% in patients with stage IB cancer (*P* = 1.000).

**Table 3:** Clinical features and their correlation with brain metastasis in patients with stage ia lung cancer

Type of analysis	*N* (%)^a^	OR	95% CI	*P* value
Clinical T stage (T1a and T1b /T1c)	1 (0.1)/10 (1.9)	21.304	2.720-166.876	0.000
Nodules (GGO component /solid)	0 (0.0) /11 (1.2)	–	–	0.002
Pathology				0.122
Adenocarcinoma	9 (0.6)	Reference	–	–
Squamous cell carcinoma	0 (0.0)	0.000	–	0.997
Other	2 (3.1)	5.086	1.076–24.038	0.040
Age (≤ 60/> 60)	6 (0.7)/5 (0.7)	1.008	0.306–3.315	1.000
Sex (male/female)	4 (0.6)/7 (0.7)	0.858	0.250–2.945	1.000
Smoking history (never /ever)	5 (0.4)/4 (1.0)	2.267	0.606–8.482	0.253
CEA (< 5 ng/ml /≥ 5 ng/ml)	4 (0.3)/1 (0.5)	1.680	0.187–15.117	0.501
CYFRA21-1 (< 3.3 ng/ml/≥ 3.3 ng/ml)	4 (0.4)/1 (0.3)	0.716	0.080–6.429	1.000
CA125 (< 35 U/ml/≥ 35 U/ml)	3 (0.2)/1 (2.0)	8.513	0.871–83.233	0.145

*CA* cancer antigen, *CEA* carcinoembryonic antigen, *CYFRA* cytokeratin 19 fragments, *CI* confidence interval, *GGO* ground glass opacity, *OR* odds ratio

^a^Percentages are based on the total number of patients with each characteristic in the study group

In patients with stage 1A lung cancer, 887 (55.6%) had primary tumors that were solid nodules and 708 (44.4%) had primary tumors with a GGO component. The solid nodules had a significant higher rate of BMs as compared with nodules with a GGO component. All 11 patients with stage 1A lung cancer and BM had solid nodules (1.2% of patients with solid nodules), and none had a GGO component (*P* = 0.002) (Table 3).

## Discussion

Due to the extensive use of MRI brain imaging prior to lung cancer resection at our institution, we were able to evaluate the value of preoperative brain MRI in a large cohort of patients with early stage lung cancer. BMs were detected in slightly less than 1% of patients with clinical stage IA lung cancer, primarily in patients with clinical stage T1c tumors. All patients with stage IA lung cancer and BM had solid lung lesions. Our study supports clinical recommendations advising against universal preoperative MRI brain imaging, but indicates that preoperative MRI may be beneficial in patients with solid T1c lung cancers. Additionally, this is the first study to identify the relationship between the incidence of BMs and lung cancer stage based on the 8th edition TNM staging system.

The incidence of BMs at initial diagnosis in patients with lung cancer is unclear. In our cohort, the incidence of BM was 5% in all patients with lung cancer, in accordance with Lee’s studies (Lee et al. 2016). The majority of BMs (87.6%) were found in patients without neurological symptoms, which is also in agreement with Lee’s results, indicating that this aggressive preoperative screening may be worthwhile to detect unexpected metastases. Lee and colleagues also reviewed the usefulness of preoperative brain MRI in patients with lung squamous cell carcinoma, and 2 out of 414 patients (0.5%) were able to avoid a futile operation due to the findings of routine brain MRI. In our study, surgery was canceled or altered to palliative resection in 105 patients (3.1%) based on brain MRI findings.

Our results confirmed that the frequency of BMs is significantly associated with clinical stage. The risk of BMs was higher in patients with advanced stage lung cancer. Our analysis of clinical stage IA patients showed only 11 BMs (0.7%). This is consistent with previous studies demonstrating that occult BMs are rare (0-0.5%) in asymptomatic, early stage NSCLC (Lee et al. 2016; Tanaka et al. 1999). In addition, previous studies demonstrated that in patients who developed BMs after potentially curative lung cancer surgery, preoperative brain MRI was not beneficial in detecting BMs and did not improve patient outcomes (Balekian et al. 2016). Thus, considering the low incidence of BMs in patients with stage IA NSCLC and the relatively high false-positive rate of brain imaging (11%), some question the use of preoperative brain MRI (Backhus et al. 2014; The Society of Thoracic Surgeons 2013). The STS and the American College of Chest Physicians recommend against brain imaging in patients with stage IA NSCLC.

There is some evidence, however, that routine preoperative brain imaging has value. O’Dowd and colleagues found that most patients with lung cancer developed metastases from early stage cancer (73% stage I or stage II)(O’Dowd et al. 2014), so they recommended that patients should have a brain MRI or head CT scan prior to surgery with curative intent, irrespective of their cancer stage. An earlier study also suggested that brain MRI was necessary in all patients with lung adenocarcinoma, because BMs were diagnosed in patients with stage I lung adenocarcinoma as frequently as in those with stage II or III lung adenocarcinoma (Park et al. 2007). Our study supports the use of preoperative brain MRI in patients whose lung cancer has progressed beyond stage IA as well as in patients with stage IA cancer that is classified as T1c (tumor size > 2 cm).

In our study, the levels of serum biomarkers, including CEA, CYFRA21-1 and CA125, were not independent predictors for BMs in multivariate analysis. A few studies have found that lung cancer patients with high serum CEA levels are at greater risk for metastatic diseases. In the study of Lee and colleagues, abnormal serum CEA levels were strongly correlated with increased whole-body metastatic potential in advanced NSCLC (Lee et al. 2014). Another study analyzed serum CEA levels in patients with stage III lung adenocarcinoma who were receiving concurrent chemoradiotherapy and demonstrated that CEA level was a predictor for BMs in these patients (Horinouchi et al. 2012). However, unlike our study, these studies did not include tumor stage as a variable when examining the role of serum biomarkers in the prediction of metastasis. It is plausible that the strong association between stage and risk of BMs that we observed might make the predictive value of serum biomarkers insignificant in our models.

Our study is the first to identify a relationship between the incidence of BMs and lung cancer stage using the 8th edition AJCC lung cancer TNM classifications, particularly the T1a/T1b/T1c criteria, during preoperative staging. The frequency of BMs was obviously higher in patients with cT1cN0 lung cancer (~ 1.9%) as compared with patients with cT1aN0 or cT1bN0 lung cancers (0.1%). Moreover, no significant difference was observed in the rate of BMs in the cT1cN0 patients when compared with patients with clinical stage IB cancer. The NCCN recommends preoperative brain MRI in patients with clinical stage IB lung cancer (National Comprehensive Cancer Network 2018). Therefore, considering the cost and yield, preoperative MRI brain imaging in patients with clinical stage IA lung cancer might not be recommended universally, but may be appropriate with risk stratification for tumor size > 2 cm.

Our results suggest that patients diagnosed as clinical T1N0 lung cancer with a GGO component in chest CT have a relatively lower risk of BMs in comparison with patients with solid nodules. Among patients with a GGO component, preoperative brain MRI showed limited value. This finding is consistent with a previous study by Cho and colleagues, who found that MRI brain imaging provides no additional staging information of pure GGO nodular adenocarcinoma (Cho et al. 2015).

This study has several limitations. First, it was a retrospective study performed at a single center. Second, post-operative complications were not analyzed, so the correlation between MRI findings and complications could not be explored. Third, PET-CT is recognized as the gold-standard method for evaluating lung cancer stage before surgery (Fischer et al. 2009); however, it is not a commonly used technique throughout the world, especially in countries, such as China, where the cost is not covered by the medical insurance system. In this study, PET-CT was not adopted as a routine preoperative staging procedure, which might limit the general applicability of our conclusions, especially at institutions where PET-CT is performed routinely. Finally, this study lacks a cost-effectiveness analysis with respect the preoperative use of brain MRI.

In conclusion, our study revealed that routine preoperative MRI brain imaging is effective for detecting BMs in patients with lung cancer. Based on the results of the study, preoperative brain MRI might help to identify BMs in patients with lung cancer that has advanced beyond stage IA. In patients with clinical stage IA lung cancer, our research suggests that routine preoperative brain MRI is not necessary but may be beneficial in patients with clinical stage T1c lung cancer (tumor > 2 cm) presenting as a solid lung lesion.

## Abbreviations

AJCC: American Joint Commission on Cancer
BMs: Brain metastases
CA125: Cancer antigen 125
CEA: Carcinoembryonic antigen
CYFRA: Cytokeratin 19 fragment
CI: Confidence interval
CT: Computed tomography
GGO: Ground glass opacity
MRI: Magnetic resonance imaging
NCCN: National Comprehensive Cancer Network
NICE: National Institute of Health and Care Excellence
NSCLC: Non-small cell lung cancer
OR: Odds ratio
PET: Positron emission tomography
STS: Society of Thoracic Surgeons
